# Production, Transport, Fate and Effects of Lipids in the Marine Environment

**DOI:** 10.3390/md23020052

**Published:** 2025-01-21

**Authors:** Christopher C. Parrish

**Affiliations:** Department of Ocean Sciences, Memorial University, St. John’s, NL A1C 5S7, Canada; cparrish@mun.ca

**Keywords:** marine lipids, ω3 polyunsaturated fatty acids, phytosterols, trophic ecology, climate change, lipidomics, phytoplankton

## Abstract

Lipids form energy storage depots, cellular barriers and signaling molecules. They are generated and metabolized by enzymes under the influence of biotic and abiotic factors, and some—the long-chain polyunsaturated ω3 and ω6 fatty acids and cholesterol—are essential for optimal health in marine organisms. In addition, lipids have direct and indirect roles in the control of buoyancy in marine fauna ranging from copepods to whales. Phytoplankton account for about half of the planet’s carbon fixation, and about half of that carbon goes into lipids. Lipids are an important component of the ocean’s ability to sequester carbon away from the atmosphere through sinking and especially after transfer to zooplankton. Phytoplankton are the main suppliers of ω3 polyunsaturated fatty acids (PUFAs) in the marine environment. They also supply cholesterol and many phytosterols to ocean ecosystems; however, genomics is indicating that members of the Cnidaria, Rotifera, Annelida, and Mollusca phyla also have the endogenous capacity for the de novo synthesis of ω3 PUFAs as well as phytosterols. It has been predicted that ω3 long-chain PUFAs will decrease in marine organisms with climate change, with implications for human consumption and for carbon sequestration; however, the responses of ω3 PUFA supply to future conditions are likely to be quite diverse.

## 1. Introduction

Almost all the water on the planet is seawater, and the oceans, with an average depth greater than 3.5 km, are home to most of the protist and especially animal biomass on Earth [1]. In addition, the marine environment harbors 31 different animal phyla, most of which contain no representatives living in the terrestrial environment [2]. In support of this biomass and biodiversity, there are about 5000 species of phytoplankton [3,4] fixing dissolved inorganic carbon into primary macromolecules and low-molecular-weight metabolites [5]. The proportions of proteins, lipids, and structural and storage carbohydrates change in response to environmental conditions, with fast-growing cells having a high demand for lipids [6].

Among the principal classes of biomolecules, lipids stand out as being large non-polymeric molecules in terms of the sheer number of distinct chemical entities. The diversity in lipid structures leads to a diversity in chemical and physical properties and then to a wide variety of biological functions, ranging from energy storage and the formation of biological membranes to cell signaling, inflammation, and immunity [7]. They are the basis of efficient storage depots, cellular barriers and extracellular and intracellular messenger molecules. They are generated and metabolized by enzymes under the influence of biotic and abiotic factors such as nutrient input and temperature.

This review describes lipid structures, their relationships and their analysis. It then examines the roles of lipids in marine ecosystems, including the production of bioactive compounds important to marine food webs as well as to humans. There is a focus on biogenic lipids, especially nutritional and biomarker lipids [8] synthesized not only by phytoplankton but by thraustochytrids, which have biotechnological applications, and by animals. However, there is an emphasis on phytoplankton as the primary producers in the oceans and also as organisms with potential for biotechnological applications. The transfer from phytoplankton though the food web and through the water column is outlined, and the potential effects of climate change are discussed.

## 2. Lipids and Lipidomics

### 2.1. Structural Diversity of Lipids

**Figure 1:**
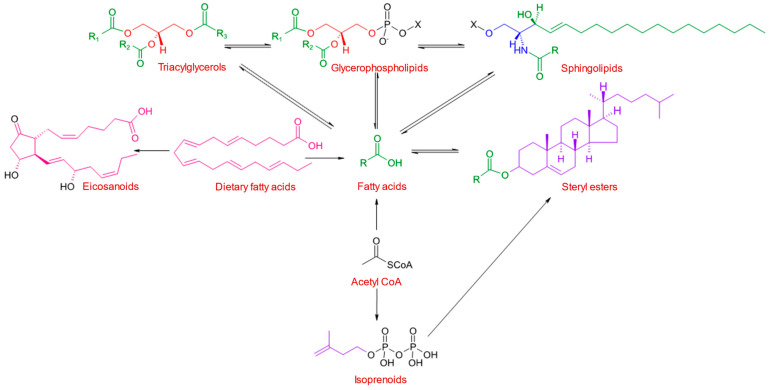
The diversity and interconnectedness of the major biogenic lipid classes. R, derived from radical, represents an alkyl group, and R-C=O (in green), an acyl group in which the total carbon chain length usually ranges from 14 to 24. Fatty acids and sterols are synthesized from the 2-carbon precursor, acetyl CoA, or are acquired dietarily. Sterols are built up from 5-carbon isoprene units (in purple). Fatty acids are esterified with glycerol (in red) in glycerolipids (the triacylglycerols and glycerophospholipids), and with sterols to form cholesteryl esters in this case. They are also transferred to be part of sphingolipids containing a serine derived group (in blue). Glycerophospholipids and sphingolipids are important membrane components, and the substituent X represents an organic base, acid or alcohol as part of the polar headgroup. Eicosanoid bioactive compounds are derived from 20-carbon precursors (eikosi is Greek for 20), in this case eicosapentaenoic acid (in magenta). This figure and legend are based on those appearing in Quehenberger et al. [10] and McDonald et al. [11].

Lipids differ from the other major groups of biomolecules (carbohydrates, proteins and nucleic acids) in that they are not polymers of relatively small numbers of different building blocks. Because lipids are operationally defined according to their extractability in nonpolar solvents, multiple subclasses of both biogenic and anthropogenic origin can be found in environmental extracts [9]. Biogenic lipid classes containing the acyl group (R-C=O) include wax esters, fatty aldehydes and free fatty acids, and they exist in highly interconnected metabolic pathways [10,11], leading to various combinations of fatty acyl chains, backbone structures, and polar headgroups (Figure 1). The variety of polar headgroups gives rise to different phospholipids, which make up about three-quarters of the total lipids in membranes [12]. These membranes form barriers for cells and organelles and act as a solvent within which membrane proteins fold and function. Each specialized membrane has a unique chemical composition leading to the variety of structures and functions [12].

### 2.2. Total Lipids, Lipid Classes and Components Versus Species Analysis

Until recently, the comprehensive analysis of lipids generally involved separation into simpler categories, according to their chemical nature. Lipids would be quantified as bulk species and/or as individual components. Bulk lipid can be determined gravimetrically, colorimetrically, or as the sum of individually measured lipid classes [13]. Lipid classes can be grouped chromatographically into acyl lipid classes, such as triacylglycerols and phospholipids, and non-acyl lipid classes, such as hydrocarbons, ketones, alcohols and sterols. With some chromatographic procedures, these classes can be isolated in order to determine the component molecules. With acyl lipid classes, a simplified approach is to determine the fatty acid composition of isolated classes or groups of classes such as neutral and polar lipids [14]. While extremely useful, this does not provide information on backbone structures or polar headgroups or how they are combined into molecular species. Advances in chromatography and mass spectrometry have permitted the identification and quantitation of lipid molecular species, which has greatly propelled our ability to study metabolic pathways and networks.

### 2.3. Characterization and Quantitation of Lipid Species in Biological Systems

Lipidomics is the comprehensive analysis of lipid molecular species, including their quantitation, in order to study metabolic pathways and networks. The large number and structural diversity of natural lipids and their modifications make lipidomic analyses challenging. It has been estimated that there may be as many as 200,000 individual lipid structures [15].

The method of choice for the sensitive detection and quantitation of lipid molecular species is mass spectrometry (MS), especially with electrospray ionization (ESI). Electrospray ionization is a “soft” ionization technique that does not cause extensive fragmentation, which is especially important for phospholipids [15]. Electrospray ionization can be applied in positive- or negative-ionization mode depending on the phospholipid class in solution. Shotgun lipidomics achieved by direct infusion allows for high-throughput analysis, but less-abundant lipid species are not detected [16]. Mass spectrometry coupled with liquid chromatography (to provide a prior separation of lipid species) increases ionization efficiency and is better suited for quantifying less-abundant molecular species.

### 2.4. Marine Lipidomics

Lipid research in natural ecosystems has focused for the most part on fatty acids with three or more double bonds [17] and to a much lesser extent on sterols [18]. However, research into lipid metabolism in marine organisms is moving toward a lipidomic approach in which comprehensive mass spectrometry-based profiling is combined with the sophisticated statistical analysis of the complex data generated (e.g., [19]). Increasingly, this approach is being used to investigate interactions between organisms and their environment, especially in the context of climate change, and this extends to past climate change events [20]. Holm et al. [21] identified 1151 distinct lipid species in suspended particulate organic matter from the surface mixed layer of several ocean basins and found highly significant negative correlations between the unsaturation levels of several plankton lipid classes and water temperature. Wood et al. [22] provide the basis for determining the lipidomic effect on the transfer of organic matter from microplankton to copepod grazers.

From a nutrition and health perspective, lipidomic analysis is also revealing bioactive lipid compounds in marine samples. For example, HPLC coupled with electrospray ionization–mass spectrometry was used to identify glycolipids and phospholipids with strong anti-inflammatory and antithrombotic properties in the cyanobacterium *Spirulina subsalsa* [23], as well as phosphatidylcholines and phosphatidylethanolamines with strong antithrombotic activities in salmon [24]. Docosahexaeonic acid-containing phospholipids from marine sources have anti-neurodegeneration and anti-cancer properties [25].

The distribution of polyunsaturated fatty acids (PUFAs) in triacylglycerol molecular species is important as well in terms of oxidative and thermal stability and absorption. Triacylglycerols provide energy storage, protective padding, insulation and buoyancy in seals, and their harvest for food holds significant cultural and traditional values for northern communities in Canada. Seal oil has long-chain PUFAs located at the *sn*-1 and -3 positions as against the *sn*-2 position in fish oil, and it lowers lipid levels in plasma and liver and reduces plasma and hepatic oxidative stress compared with fish-oil feeding [26].

### 2.5. Trophic Ecology of Lipids

The characterization of energy flow in ecosystems is one of the main goals of ecology, and the analysis of trophic interactions and food web dynamics is key to quantifying its flow [27]. Lipids are central to these studies, yielding more than twice the amount of energy per gram than proteins or carbohydrates and providing essential fatty acids and sterols, as well as a means to quantify trophic connections through biomarker analysis. Thus, the determination of the origin, abundance, and transfer of marine lipids will aid in the effective monitoring of the sustainability and health of marine ecosystems. To accomplish this depends on research on the effects of essential fatty acids (EFAs) on carnivores and omnivores in natural systems, including whether EFA limitation affects food web structures or biogeochemical processes [17]. Aquatic organisms need certain PUFAs to support optimal health, and there are several displaying essential-fatty acid activity [28], such as linoleic acid (LIN, 18:2ω6), arachidonic acid (ARA, 20:4ω6), ω6 docosapentaenoic acid (ω6DPA, 22:5ω6), and especially eicosapentaenoic acid (EPA, 20:5ω3), and docosahexaenoic acid (DHA, 22:6ω3).

The term EFA has become shorthand for rather more precise and accurate terminology that distinguishes between essential metabolites and essential nutrients [29] and whether they are conditionally indispensable or conditionally dispensable [30]. In marine organisms, there are differences within phyla ranging from protists to zooplankton to fish [28,31,32], which genomic approaches are helping unravel [32].

In parallel with nutritional considerations, fatty acid profiling is used to examine trophic interactions in terrestrial and marine systems [33,34], including the use of compound specific isotope analyses. Fatty acid profiling can also be used to indicate anthropogenically induced dietary shifts from aquatic to terrestrial foods [35].

Sterols, which have been much less studied than fatty acids in aquatic ecosystems, can be co-limiting nutrients together with EFAs in freshwater herbivores [36]. Sterols control membrane fluidity and permeability in all eukaryotic cells, and the ordering capacity provided by cholesterol is different to that provided by other sterols [37]. Besides cholesterol, there are about 250 phytosterols, all of which regulate membrane fluidity but with different efficiencies. Sitosterol and campesterol are the most efficient of the phytosterols [38]. However, a lower conformational ordering effect compared to cholesterol [39] may limit their use in most animals [37].

In parallel with nutritional considerations, sterol profiling has been used in sediments collected from lacustrine, estuarine and marine environments to trace changes in organic matter inputs over time [40]. In addition, sterol analyses can be used to track anthropogenic inputs using coprostanol and epicoprostanol, which are specific for sewage [40]. Although fish, crustaceans, and cephalopod mollusks contain cholesterol as their predominant sterol (often >90% of sterols), the sterol profiling of invertebrates in other taxa can be used in trophic ecology studies [41]. Some invertebrates have twenty or more different sterols, and some, such as bivalve mollusks or sponges, may have a sterol other than cholesterol as the major constituent [42].

## 3. Lipids in Marine Ecosystems

### 3.1. High Carbon and Hydrogen Content

Lipids are mainly carbon—for example, one of the most common fatty acids in the biosphere, oleic acid (18:1ω9), with the chemical formula C_18_H_34_O_2_, is 77% carbon by mass, versus 41% for glutamine and 40% for glucose. Being largely composed of carbon and hydrogen, lipids have more than twice the specific energy (kJ/g) of proteins or carbohydrates. Also, being carbon- and hydrogen-rich, they are hydrophobic and can act as a solvent and absorption carrier for organic contaminants and thus can be drivers of pollutant bioaccumulation in marine ecosystems.

### 3.2. Indispensable Nutritional Molecules

Among the lipids, long-chain (LC) polyunsaturated ω3 and ω6 fatty acids and cholesterol are essential for survival and normal somatic growth in marine organisms, supplying structure to cell membranes and precursors of metabolic messengers, and they play an integral role in immunity [18]. While some organisms can synthesize these compounds from precursors [43], many have a limited ability and so have to be supplied in the diet. This extends to humans: an analysis of blood from over 40,000 adults revealed that ω3 LC-PUFA intake was associated with a lower risk of premature death [44]. These PUFAs have beneficial effects on cardiovascular disease, mental illnesses, age-related cognitive decline, periodontal disease, and rheumatoid arthritis [45]. A cohort study revealed an inverse association between intake of ω3 LC-PUFAs and death due to respiratory diseases and with total mortality [46]. Aquatic foods are the key source of these essential ω3 LC-PUFAs, and aquaculture is expected to meet the rising global demand for these foods [47]. Global trade in aquatic products continues to grow, with salmon being the most valuable traded aquatic animal product [47].

A well-known property of the ω3 C_20_ fatty acid EPA is that it provides an alternative eicosanoid substrate to the ω6 C_20_ fatty acid ARA. Eicosanoids are oxidized derivatives of 20-carbon polyunsaturated fatty acids, acting on many cell types and affecting inflammation and immunity, and arachidonic acid is the usual substrate for eicosanoid synthesis [48]. However, the increased incorporation of EPA in membranes would be at the expense of ARA, and EPA results in the production of less-potent eicosanoids. C_22_ DHA, on the other hand, plays a crucial regulatory role in the resolution of acute inflammation through oxidation to specialized pro-resolving mediators (SPMs). Three families of mediators, termed “resolvins” (for resolution phase interaction products), “protectins”, and “maresins” (for macrophage mediators in resolving inflammation), act in the pico- to nanomolar range [49]. DHA is enzymatically oxidized to add one to three hydroxyl groups (-OH) by cyclooxygenases (COXs) and lipoxygenases (LOXs) to produce mono-, di-, and trihydroxy DHAs, respectively. Monohydroxy DHA is involved in wound healing and is the precursor for the dihydroxy DHAs found in D-series resolvins, protectins and maresins and the trihydroxy DHAs in D-series resolvins [50,51]. There are also bioactive molecules with potential pro-resolving activity derived from the oxygenation of EPA: the E-series resolvins [50]. Both D- and E-series resolvins are reported to be key lipid mediators in the diverse resolution of the inflammatory process [50].

Thus, overall EPA has an anti-inflammatory effect through the combination of replacing ARA in membranes [52], through the release of eicosanoids with only weak proinflammatory activity [53] and through the generation of resolvins with anti-inflammatory actions [52]. DHA, however, has been found to be more effective than EPA in modulating markers of inflammation and lipid risk factors [54]. In addition to providing pro-resolving mediators, DHA is found in high concentration in signaling systems [55]. It is the principal structural component of the visual system, synapses and neurons in animals, and DHA from the marine food web is thought to have played a critical role in human evolution [55]. The cohort study of Wang et al. [46] showed that DHA intake was associated with lower total mortality.

Sterols are major lipid components of membranes in most organisms. They are planar, rigid tetracyclic molecules used by fungi, plants and animals to modulate membrane fluidity, permeability and function [37]. Ergosterol is the major sterol in the membranes of yeast and fungi, and cholesterol dominates in animals and especially arthropods, cephalopods, fish and mammals. In between, algae have a wide array of phytosterols, which have been investigated for their human health benefits associated with their antioxidant, anti-inflammatory, immunomodulatory and cholesterol-lowering properties [56]. They provide an abundance of benefits through their interaction with enzymes and various other proteins actively participating in different cellular pathways [56].

Some marine invertebrates require dietary sources of sterols for growth and survival because of their inability to synthesize them de novo. Crustaceans, nematodes, some mollusks and some ciliates require dietary sources [37]. Herbivores either use the sterols present in their diets directly or metabolize them to cholesterol to meet requirements for growth and development. However, the capacity to convert dietary phytosterols into cholesterol differs among phytosterols as well as among species [37]. Marine invertebrate sterols and derivatives have antiviral, antibacterial and antifungal activities [57].

### 3.3. Buoyancy, Thermoregulation, Echolocation

An important but sometimes overlooked aspect of lipids is their direct and indirect roles in the control of buoyancy in marine animals. Triacylglycerols have a density of 0.93 g/mL and wax esters an even lower one at 0.86 g/mL; this gives them a much larger buoyancy of 0.165 g/mL, compared to 0.095 g/mL for triacylglycerols, in seawater of density 1.025 g/mL [58]. Many fish in the oceans have triacylglycerol in their bones, providing indirect buoyancy, as the primary role would be energy storage. Some fish, however, appear to use wax esters primarily for buoyancy [58].

In marine mammals, blubber is used for buoyancy adjustment, insulation, energy storage, metabolic water, streamlining and as an elastic spring [59]. Blubber usually contains primarily triacylglycerols, but wax esters dominate in toothed whales: the beaked and sperm whales [59]. Specialized fat bodies comprising wax esters and unusual triacylglycerols exist in toothed whale heads for hearing and echolocation [60]. Wax ester is the dominant lipid storage class in pelagic animals associated with deep-water habitats: copepods, deep-water and vertically migrating fish, and toothed whales. Buoyancy can be controlled in such organisms by exploiting the phase transition of wax esters from liquid to solid and back again, in the process contracting and becoming denser and then reversing the contraction. This reversal is accomplished by reheating in deep-diving whales [61] and by selectively catabolizing polyunsaturated wax esters in diapausing copepods [62]. The poikilothermic copepods have high levels of polyunsaturated wax esters in which solid–liquid phase changes are primarily controlled by pressure [63]. Catabolism of the denser components of the lipid pool while overwintering would aid with re-ascent to surface waters at the end of diapause.

### 3.4. Alterations with Climate Change

Climate change is exposing ecosystems to unprecedented changes in physical and chemical characteristics, which individually and together are affecting all life in the ocean [64]. Canada has the longest coastline in the world and all of its oceans are warming, while pH is decreasing [65,66], especially in the Arctic [67]. These aspects, singly and in combination, constrain and enhance different aspects of the flow of carbon and energy through food webs [68,69]. The quantity, quality, and dynamics of lipids within organisms and ecosystems are expected to alter with climate change, with consequences for the flow of energy [68] and contaminants, especially lipophilic persistent organic pollutants [70]. Such effects extend to the zooplankton-mediated transport of phytoplankton-derived carbon to the deep ocean [71,72], which itself affects the global carbon cycle [73].

In the northern hemisphere, temperature is the principal factor affecting the trophic structure of marine ecosystems through both direct and indirect mechanisms [74], and this may be exacerbated by the concomitant decrease in pH. Ocean acidification is widely distributed and will influence many regions, including planktonic systems, benthic environments and the deep sea [75]. Human-driven ocean acidification affects organism-level physiology, biomineralization, growth, and reproduction [76]. For example, Rossoll et al. [77] hypothesized that projected drops in mean oceanic surface pH could have indirect effects on zooplankton growth through the alteration of the biochemical composition of phytoplankton. They analyzed direct and indirect effects of increasing CO_2_ concentrations on a diatom–copepod system focusing on fatty acids, somatic growth and reproduction. Copepod egg hatching success is high in ecosystems where diatoms dominate around the world, and copepods are prey for larval fish in some of the most productive ecosystems in the oceans [78]. In the acidified copepod–diatom system [77], saturated fatty acids increased and PUFA proportions decreased in both copepods and their food, and consumer growth and egg production also decreased. The increase in saturated fatty acids was likely a membrane response to lowered pH, which would be exacerbated by increased temperature [79].

### 3.5. Marine Lipids and Trophic Connections

For more than half a century, fatty acids have been used to determine trophic connections in the oceans. In 1965, Ackman and Eaton [80] noted the similarity in fatty acid profiles obtained from whale and krill samples, especially in terms of the high proportions of the long-chain monoenes 20:1 and 22:1, and they concluded that there was a direct deposition of dietary fatty acids. This foreshadowed the continually expanding practice of identifying key source fatty acids and comparing fatty acid profiles in marine consumers and their diets. Fatty acid profiling can also be used to indicate chemical stress in marine organisms [81].

Tabulated profiles of fatty acid data can be compared visually, but obviously, these kinds of data lend themselves well to multivariate statistical analyses. For example, in a study of fatty acid analyses of skipjack tuna and albacore tuna from different locations in the southwest Pacific, the skipjack-tuna fatty acid profile was found to have the highest similarity with krill (at 74%), followed by Tasmanian albacore tuna (with 65% similarity) [82] using similarity of percentages analysis (SIMPER). This can be compared with salmon fed known diets where there was a narrow range of 86–88% similarity between fatty acids in muscle tissues and paired dietary composition (Appendix A). These values obtained from aquaculture feeding trials with well-characterized diets [83] can be used to calibrate the similarity measures from the field data [82]. If the ~13% dissimilarity in the feeding trial (Appendix A) applied to the tuna data, then 74 × 100/87 = 85% of skipjack dietary fatty acids would be derived from krill fatty acids, and for the Tasmanian albacore it would be 65 × 100/87 = 75%. Tuna feeding experiments would be needed to verify these calculations, and there may be an opportunity to execute this in South Australia where there is considerable net-pen aquaculture of bluefin tuna.

The very simple approach of statistically matching the fatty acid profiles of consumers and their diets can be refined by focusing on neutral lipids in the consumer [84,85]. Operationally defined neutral and polar lipids have different functions and structures, with neutral lipids predominantly consisting of storage triacylglycerols and wax esters and polar lipids mainly comprising membrane glycolipids and phospholipids. As a result, the type of fatty acid incorporated into polar lipids is more limited, while fatty acid deposition into storage reflects the dietary composition because the fatty acids themselves are not degraded during digestion [86]. The ultimate aim of comparing fatty acids in consumers and their diets is not just simply to quantify the degree of similarity but to estimate the amount of different dietary sources consumed. Quantitative fatty acid signature analysis (QFASA) does this for high-trophic-level predator–prey relationships using predator adipose-tissue composition [86]. Over a couple of decades, this approach has continued to be refined and applied to increasing numbers of species, including those at lower trophic levels [87].

Sterols are also excellent biomarkers due to their stability in addition to the diversity of their structures, and marine crustaceans and bivalves require a dietary supply for somatic growth [37]. The use of multiple tracers in marine ecology [34,88] is particularly powerful, and now, mass spectrometry-based lipidomic analyses have permitted the detection of over a thousand individual lipid species across several ocean basins [21].

## 4. Phytoplankton Lipids

Phytoplankton account for about half of the world’s annual carbon fixation, leading to annual global stocks of carbohydrate, protein and lipid of 0.04, 0.17 and 0.11 gigatons, respectively [89]. Using the carbon content of 40% for glucose, 41% for glutamine and 77% for oleic acid, it can be estimated that lipids account for most (~49%) of the phytoplankton carbon. Also, with lipids having more than twice the specific energy of proteins or carbohydrates, it can be estimated that lipids account for over half (~53%) of the phytoplankton energy. In addition to carbon and energy, phytoplankton lipids include indispensable nutritional molecules for food webs such as essential fatty acids and sterols [18].

### 4.1. Main Suppliers of ω3 Polyunsaturated Fatty Acids in Marine Ecosystems

**Figure 2:**
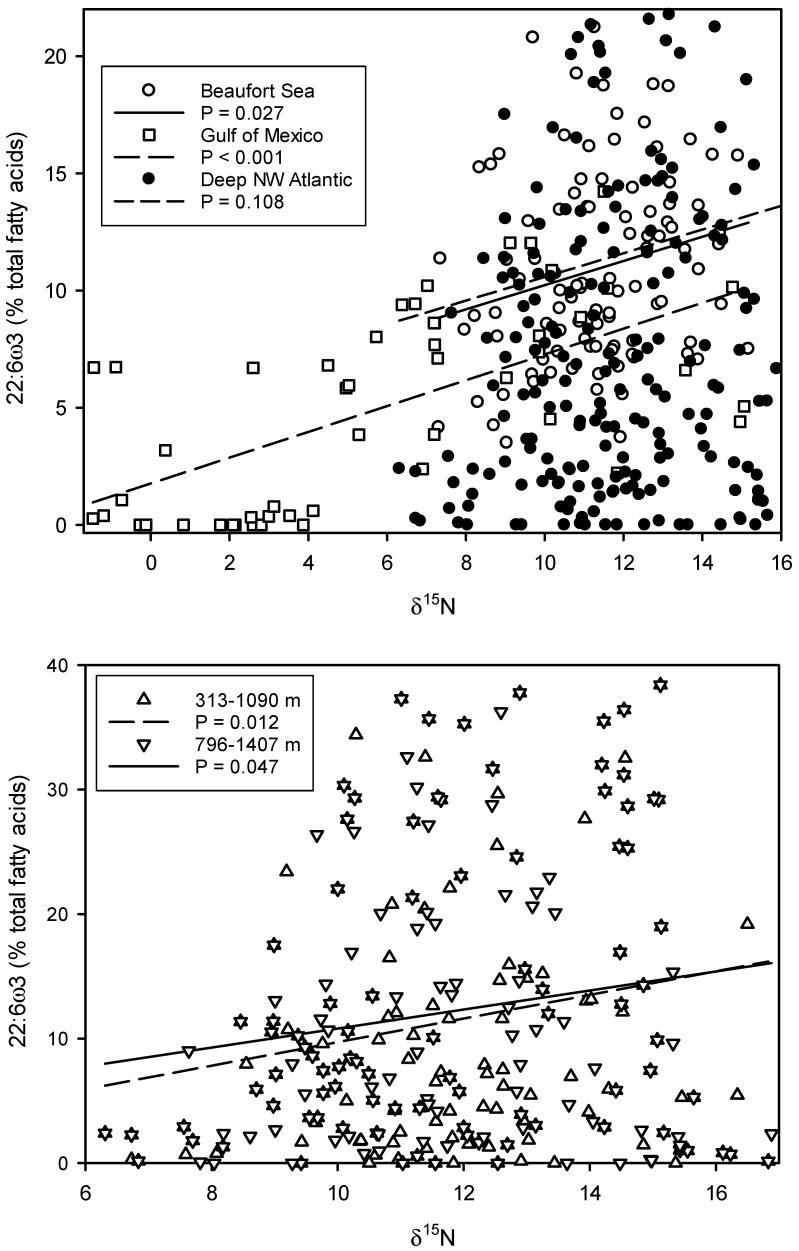
Trophic magnification of DHA in marine food webs from three very different environments: the Beaufort Sea Shelf [92], the southwest Gulf of Mexico [94] and the continental margin in the northwest Atlantic [95]. DHA proportions are plotted against bulk nitrogen stable isotope ratios (δ^15^N) as a proxy for trophic position. The bottom panel shows two separate significant regressions according to depth in the northwest Atlantic on a different scale.

Phytoplankton are the main suppliers of ω3 PUFAs in marine ecosystems [90], with eustigmatophytes and diatoms supplying the highest amounts of EPA per unit carbon and haptophytes and dinoflagellates supplying the most DHA. Phytoplankton long-chain PUFAs are a key component of food quality, affecting patterns of energy and nutrient flow at the primary producer–herbivore interface [91]. These PUFAs are transferred with little alteration through the food web, and one or the other [92,93] and sometimes both [94] have been shown to be biomagnified across trophic levels in very different environments. This suggests the preferential faunal assimilation and/or preferential oxidation of other fatty acids. The data in Figure 2 show remarkable parallel increases in DHA proportions with increasing δ^15^N, a proxy for trophic level. Two of the increases are significant [92,94] and one is not [95]. However, the latter samples came from a wide depth range: 313–1407 m depths over shelf and slope areas off the Canadian province of Newfoundland. Using different depth ranges, two separate significant regressions were obtained with deeper, lower trophic-level samples possessing higher proportions of DHA, perhaps as a homeoviscous adaptation to pressure [96].

Increases in DHA proportions of about 1% per trophic level (Figure 2) suggests the preferential faunal assimilation of DHA and/or preferential removal of non-EFAs. It is thought that maintaining a supply of marine DHA will play a key role in the future health of mankind [55], which underlines the need for studies of mismatches in ω3 PUFA supply and demand within and between ecosystems [17]. Mismatched PUFA production at the base of the food web would affect higher trophic consumers, including high-commercial value-species and humans. The latitudinal shift in the regression lines suggests a temperature-based shift in DHA production at the base of each food web in response to changes in species or a membrane structural response to temperature change. The proximity of the regression lines from the cold-water samples emphasizes the importance of temperature.

The transfer of long-chain PUFAs with little alteration through the food web, and the limited ability of other organisms to synthesize them, has formed the basis of their use as biogeochemical markers to trace the spatial and trophic ecology of marine animals [34]. However, polyketide synthase-derived PUFAs in thraustochytrids and some marine bacteria [97], as well as genes for ωx desaturase-derived PUFAs in 80 species of invertebrates [98], suggest there may be other important suppliers of ω3 PUFAs in marine ecosystems. This, combined with biomagnification (Figure 2), would also limit their use as biomarkers, although PUFAs cannot be synthesized de novo by vertebrates or other chordates [99], and, in any case, many other fatty acid biomarkers are available [100,101,102,103].

### 4.2. Sterols

Microalgae also supply cholesterol and over 30 phytosterols to marine ecosystems [104], and the algal synthesis of some sterols is sensitive to growth temperature [105]. Sterols control membrane fluidity and permeability in eukaryotic organisms [37], and cholesterol and stigmasterol stimulate the export of H^+^ at low concentrations, whereas all other sterols act as inhibitors [38]. This could be important in the context of ocean acidification (OA) as the study of Rossoll et al. [77] suggests increased amounts of saturated fatty acids are used to control internal cell pH by making membranes less fluid and permeable to CO_2_.

Phytoplankton sterols have been detected in sponges, cnidarians, mollusks, crustaceans, and echinoderms collected on the continental slope of the northwest Atlantic [106]. This would indicate that a phytoplankton-derived food supply reaches deep-water benthic invertebrates, which is important given the limited ability of invertebrates to synthesize sterols, even from other sterols [107]. However, some annelids are capable of synthesizing phytosterols de novo, and this capability may extend to some members of other phyla [108].

### 4.3. Microalgae and Aquaculture

Microalgae grow in freshwater, seawater or even wastewater to contain up to 75% lipid, up to 80% protein, up to 58% carbohydrate, and EPA at up to 34% of fatty acids, depending on species and growth conditions [109]. Feeding microalgae is important in bivalve, fish, shrimp, sea cucumber and crab aquaculture, particularly during the larval and juvenile stages [110,111,112]. These photosynthetic microorganisms can be added to the rearing water for direct feeding by invertebrates and as an initial prey for fish larvae [111], as well as to initiate green water [111] and biofloc systems [113]. Microalgae biomass can also be fed as a paste or form the basis of a pelleted feed [114].

Microalgae such as the flagellate *Pavlova* sp. (Pav, CCMP 459) can be cultivated in large-volume photobioreactors to produce high-quality food for invertebrates and fish [115]. In studies of binary microalgal diets, this prymnesiophyte and the centric diatom *Chaetoceros muelleri* provided the best growth rate for scallop larvae [116], postlarvae [110], and juveniles [117]. The results indicated that DHA and the long-chain ω6 PUFAs ARA and ω6DPA were essential for optimizing the growth of early life stages of sea scallops, *Placopecten magellanicus,* and bay scallops, *Argopecten irradians*. *C. muelleri* is a good source of ARA for *P. magellanicus* [118], while *Pavlova* sp. (Pav, CCMP 459) had 7.2% [119] to 8.9% [116] ω6DPA depending on growth conditions. C_22_ ω6DPA was readily taken up by phospholipids in salmon liver and muscle on feeding *Pavlova* biomass [119]. It seems that ω6DPA is an overlooked essential fatty acid in aquatic food webs [28]. Cholesterol provided by *Chaetoceros muelleri* may have also contributed to the high growth rates [120,121]. In addition to ω6DPA, *Pavlova* sp. (Pav, CCMP 459) is also a source of cholesterol [119].

### 4.4. Algal Biotechnology

The large-scale cultivation of algae, both microalgae [122] and macroalgae [123], can be used for carbon capture and the provision of phytochemicals. For example, macroalgae provide a variety of bioactive compounds, including polysaccharides, with activity against toxic bacteria [124]. They also provide glycolipids with antimicrobial, antitumor, antifungal and anti-inflammatory activity [125]. Some seaweeds can be used for direct human consumption, providing lipids and essential fatty acids: edible macroalgae contained lipids at up to 6.2% dry mass [126], ARA at up to 27% of fatty acids, 18:3ω3 at up to 25% of the fatty acids [127], and EPA at up to 22% of fatty acids [126].

In a listing of 34 industrial sources of EPA and DHA, the highest individual amounts occur in the microalgae with up to 4% dry mass of EPA and 2.7% dry mass of DHA [128]. The inclusion of algae, both microalgae and macroalgae, in aquafeeds is known to have beneficial effects on aquatic animals [112]. Their cultivation can also contribute to the treatment of wastewater and waste gas [109,129,130].

Algal enzymes have also been investigated for the genetic engineering of oilseed crops to produce fish-oil levels of ω3 LC-PUFAs with a high ω3/ω6 ratio [131]. Photosynthetic green algae and the haptophyte *Pavlova salina* have been used to isolate desaturases (Δ4, 5, and 6) and elongases (Δ5 and 6) to develop a genetically engineered canola that produces a DHA-containing oil [131].

## 5. Polyketide Synthase-Derived PUFAs

### 5.1. Pathway Has Been Identified in Bacteria and Thraustochytrids

Most long-chain PUFAs are synthesized through the alternate use of desaturases and elongases, but there are also pathways of PUFA synthesis that do not require the aerobic desaturation and elongation of saturated fatty acids. Anaerobic polyketide synthase (PKS) systems conduct the same reactions but in an abbreviated sequence [99]. This pathway has been identified in thraustochytrids and cold-water bacteria [97]. PUFA synthesis by bacteria in the deep ocean, sediments and fish intestines [132] could represent an important EFA subsidy in the face of reduced production by phytoplankton caused by global change. Compound-specific isotope analyses can be used to detect PKS-derived PUFAs because they are ^13^C-enriched as a result of fewer synthetic steps causing less kinetic fractionation [28].

### 5.2. Thraustochytrid Biotechnology

Thraustochytrids have even higher amounts of DHA than can be found in algae: 18% dry mass of DHA [133]. Such remarkably high amounts are achievable from oil contents in the 50–77% dry mass range with DHA > 30% of the fatty acids in the oil produced [134]. Using thraustochytrids, it is possible to investigate the dietary effects of DHA while minimizing dietary EPA, and fish feeding studies have suggested a preference for DHA over EPA [135]. This is important because DHA affects membrane physico-chemical properties, intracellular signaling pathways and gene expression differently. In addition, the production of thraustochytrids can contribute to the treatment of wastewater, further enhancing their sustainability [135].

## 6. ω3 Long-Chain PUFAs and Climate Change

### 6.1. ω3 LC-PUFAs Predicted to Decrease in Organisms

Temperature is the major factor exerting trophic control in marine ecosystems [74], and the food web supply of PUFAs could be one of the reasons. ω3 LC-PUFA supply from phytoplankton may be reduced with increasing temperature in response to changes in species [136] or as a membrane structural response to temperature change [137]. A reduction in PUFA production at the base of the food web would then propagate through other trophic levels.

ω3 LC-PUFAs are predicted to decrease in a variety of organisms as a result of cellular membrane adaptation to warming waters, not only through reduced trophic transfer from plankton [21,138] but also through their own homeoviscous response to temperature [139,140]. Temperature-induced declines could be exacerbated by ocean acidification, further reducing the availability of seafood ω3 LC-PUFAs for human consumption [138], with potential impacts for carbon transfer to deep water. The seasonal copepod lipid pump [71] is only second to the mesopelagic-migrant pump among the particle-injection pumps sequestering carbon away from the atmosphere [72]. The ability of wax esters to undergo a phase transition under pressure depends on the level of unsaturation [63], so a significant reduction in dietary PUFAs may limit the ability of copepods to be neutrally buoyant at depth.

### 6.2. ω3 LC-PUFA Response to Future Conditions Is Complex

Recent studies have underlined the complexity of the ω3 LC-PUFA response to future conditions, especially when environmental drivers are combined [141]. For example, stratification-induced nitrogen limitation in the Arctic will likely increase lipids and PUFAs in sea-ice microalgae [142]. The effect of temperature itself on ω3 LC-PUFAs may depend on the length of exposure and species, in addition to temperature range. For example, cultured diatom species responded differently to 1-week and 2-year warming, and most were able to restore PUFA contents after long-term warming [143]. A study with sea bass actually showed increased DHA at higher temperature, as well as improved growth, even with a decreased availability of dietary ω3 LC-PUFAs [144]. EPA+DHA contents of wild fish do decrease with increasing temperature, but only over an intermediate and narrow temperature range [145]. The seasonality of biological and physical interacting processes may be important too: Antarctic krill fatty acids and sterols showed little response to increased *p*CO_2_ levels until they reached extremely high values, but this was not in all seasons [146]. To help unravel the complexities, there are calls to measure fatty acid unsaturation on daily to seasonal time scales [21]. There are also calls to measure fatty acid contents per weight (mg g^−1^) rather than proportions of total fatty acids (% total fatty acids) in order to determine nutritional value [145]. Additionally, researchers emphasize differences in phospholipid fatty acids and those in reserve neutral lipids (e.g., triacylglycerols) [145]. Finally, it will be important to investigate non-phytoplankton sources of ω3 LC-PUFAs as these may compensate for any deficiency in phytoplankton supply [43]. In addition to bacteria and thraustochytrids, invertebrates in several phyla have the ability to produce ω3 PUFAs de novo [98]. These invertebrates have ωx desaturases that introduce a double bond between an existing double bond and the methyl end of the fatty acid. It is essential we establish which members of the Cnidaria, Rotifera, Mollusca, Annelida, and Arthropoda phyla undertake the de novo synthesis of ω3 PUFAs and under which conditions.

## 7. Conclusions

The determination of the origin, abundance, and transfer of lipids will aid in effectively monitoring the sustainability and health of marine ecosystems. Lipids are central to the characterization of carbon and energy flow in marine ecosystems, and the analysis of trophic interactions and food web dynamics is key to quantifying its flow. Lipids supply over twice the amount of energy than proteins or carbohydrates, as well as essential fatty acids and sterols. Their chemical determination provides an array of compounds that can be used to quantify trophic connections. Organismal lipid content is sensitive to temperature and pH and may be used to assess effects of climate change on marine ecosystems. A key area for further study includes the effects of environmental changes, such as warming and ocean acidification, on lipid production and composition across trophic levels. Measuring concentrations in storage lipids and membrane lipids separately will greatly aid in distinguishing dietary from environmental responses in marine organisms. Comprehensive quantitative analyses of intact lipid molecular species will further unravel these responses as well as uncover new bioactive lipid compounds. There are many more marine organisms yet to be revealed, each with potential for the discovery of compounds suitable for drug production. Combining lipidomics and genomics will be important to further delineate the production, transport, fate and effects of lipids in the marine environment, especially in the context of climate change.

## Figures and Tables

**Figure A1:**
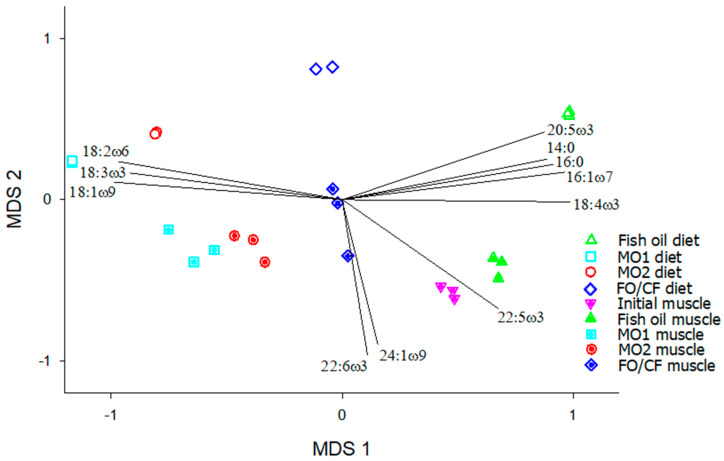
MDS plot of untransformed fatty acid proportions in cultured salmon and their diets [83]: model oils (MOs) and fish oil and chicken fat (FO/CF). The resemblance matrix was created using Bray–Curtis similarity coefficients. Axes are Pearson correlations with the variables. Vectors with Pearson correlations >0.90 are shown for fatty acid data obtained from aquaculture feeding trials [83]. Also, 2D stress = 0.02.

## Data Availability

Not applicable.

## Appendix A. Multivariate Analysis of Trophic Similarity in Marine Fatty Acid Profiles

The advent of direct transesterification methods on freeze-dried [147] or even wet samples [82] obviates the necessity of the laborious lipid-extraction step. The rapid throughput of tissue fatty acid analyses permits the generation of large fatty acid data sets, which lend themselves well to multivariate statistical analysis. In a very simple example, PRIMER V7, a software package for analyzing multivariate ecological data, was used to measure similarity within and between sample groups of albacore tuna, skipjack tuna, and krill fatty acid proportions [82]. PRIMER was also used to provide simple graphic representations of the data set based on non-metric MDS analysis. Within-group similarities ranged from 76 to 93%, and between-group similarities ranged from 59 to 78% [82]. The question remains: What do these similarities actually imply? To help answer this question, these values were compared here with those obtained from data from aquaculture feeding trials with well-characterized diets in order to calibrate the similarity measures from the field data.

The same statistical routines were used here on published data for salmon aquaculture feeding trials [83] using diets with a wide range of ω3 and ω6 fatty acid proportions. The fatty acid data were collected in the same laboratory on the same gas chromatograph with the same column. The purpose of these analyses was to compare and quantify statistical parameters derived from controlled feeding studies with those produced by generalist predators collected in situ. Through clarifying such values, we can start to understand how to better quantify SIMPER-derived feeding relationships.

Figure A1 shows a two-dimensional configuration plot of an MDS analysis of a resemblance matrix of fatty acid data from the salmon feeding trial that was performed with very different diets [83]. The vectors in this MDS plot and that for the tuna fatty acid data [82] show the importance of the essential fatty acids DHA and EPA in determining similarities among samples in these two completely different studies. In the MDS plot for the salmon feeding, both DHA and EPA gave Pearson correlations of 0.97.

The Σω3 and the Σω6 fatty acids in the diets varied two- to three-fold, while the DHA/EPA ratio and the Σω3/Σω6 fatty acid ratio varied five- to six-fold [83]. This resulted in a very large range of similarities (58–90%) in comparisons between salmon muscle samples and the various diets (Table A1). However, when muscle samples are compared with the actual diet fed, the range is much smaller (2%) and the similarity is high (>86%). The within-group similarity was always very high (>96%) and, as with the southwestern Pacific albacore tuna and skipjack tuna fatty acid data [82], 16:0 and DHA and/or EPA were always among the top four contributors to the similarity in each of the eight groups, contributing 9–21% each.

Hall et al. [148] used SIMPER to compare the average within-group and between-group similarities, usually terming ≥90% as ‘high’. By this measure, the within-group similarity of diets and muscles in Table A1 would be very high. In the salmon fed known diets, there was a narrow range of 86–88% similarity between fatty acids in muscle tissues and paired dietary composition. If we take 87% as an average similarity, or 13% as dissimilarity, then a re-analysis of data from the aquaculture experiment suggests an amendment to the old adage ‘you are what you eat’. In this experiment, one might say ‘you are what you eat plus ~13% biochemistry’.

**Table A1:** SIMPER values for untransformed fatty acid proportions in salmon and their diets in an aquaculture feeding trial [83]. The diets contained model oils (MO) or fish oil and/or chicken fat (FO/CF). PERMDISP test for homogeneity of multivariate dispersions F = 4.08, *p* = 0.084. ANOSIM Global R statistic = 0.995, *p* = 0.1%.

Average Similarity Within/Between Groups (%)								
	Fish Oil Diet	MO1 Diet	MO2 Diet	FO/CF Diet	Fish Oil Muscle	MO1 Muscle	MO2 Muscle	FO/CF Muscle
Fish oil diet	**99**							
MO1 diet	58	**99**						
MO2 diet	65	92	**99**					
FO/CF diet	77	75	83	**98**				
Fish oil muscle	**86**	62	68	78	**97**			
MO1 muscle	65	**87**	89	82	73	**96**		
MO2 muscle	69	82	**87**	85	78	94	**97**	
FO/CF muscle	77	75	81	**88**	84	86	90	**96**

Within group and related between group similarities are bolded.
